# Macrophage CARD9 mediates cardiac injury following myocardial infarction through regulation of lipocalin 2 expression

**DOI:** 10.1038/s41392-023-01635-w

**Published:** 2023-10-13

**Authors:** Yan Liu, Yi-hui Shao, Jun-meng Zhang, Ying Wang, Mei Zhou, Hui-qin Li, Cong-cong Zhang, Pei-jie Yu, Shi-juan Gao, Xue-rui Wang, Li-xin Jia, Chun-mei Piao, Jie Du, Yu-lin Li

**Affiliations:** Beijing Anzhen Hospital, Capital Medical University; The Key Laboratory of Remodeling-Related Cardiovascular Diseases, Ministry of Education; Beijing Collaborative Innovative Research Center for Cardiovascular Diseases; Beijing Institute of Heart Lung and Blood Vessel Diseases, Beijing, 100029 China

**Keywords:** Cardiovascular diseases, Inflammation

## Abstract

Immune cell infiltration in response to myocyte death regulates extracellular matrix remodeling and scar formation after myocardial infarction (MI). Caspase-recruitment domain family member 9 (CARD9) acts as an adapter that mediates the transduction of pro-inflammatory signaling cascades in innate immunity; however, its role in cardiac injury and repair post-MI remains unclear. We found that *Card9* was one of the most upregulated *Card* genes in the ischemic myocardium of mice. CARD9 expression increased considerably 1 day post-MI and declined by day 7 post-MI. Moreover, CARD9 was mainly expressed in F4/80-positive macrophages. *Card9* knockout (KO) led to left ventricular function improvement and infarct scar size reduction in mice 28 days post-MI. Additionally, *Card9* KO suppressed cardiomyocyte apoptosis in the border region and attenuated matrix metalloproteinase (MMP) expression. RNA sequencing revealed that *Card9* KO significantly suppressed lipocalin 2 (*Lcn2*) expression post-MI. Both LCN2 and the receptor solute carrier family 22 member 17 (SL22A17) were detected in macrophages. Subsequently, we demonstrated that *Card9* overexpression increased LCN2 expression, while *Card9* KO inhibited necrotic cell-induced LCN2 upregulation in macrophages, likely through NF-κB. *Lcn2* KO showed beneficial effects post-MI, and recombinant LCN2 diminished the protective effects of *Card9* KO in vivo. *Lcn2* KO reduced MMP9 post-MI, and *Lcn2* overexpression increased *Mmp9* expression in macrophages. Slc22a17 knockdown in macrophages reduced MMP9 release with recombinant LCN2 treatment. In conclusion, our results demonstrate that macrophage CARD9 mediates the deterioration of cardiac function and adverse remodeling post-MI via LCN2.

## Introduction

Heart disease is the main cause of death worldwide,^1^ and acute myocardial infarction (MI) is a common cardiovascular disorder leading to necrosis of cardiomyocytes. Owing to lack of regenerative potential of the adult heart, damaged myocardium is replaced by scar, which adversely affects cardiac function. Consequently, patients who survive an MI often experience heart failure. Efforts to reduce cardiac injury or facilitate repair post-MI may help to prevent heart failure. Innate immunity is elicited and plays an essential regulatory role post-MI in response to danger-associated molecular patterns (DAMPs) released by dead myocytes.^2^ Immune cells are recruited to amplify inflammation. Monocyte infiltration into the infarcted heart starts soon after MI via C-C motif chemokine ligand2 / C-C motif chemokine receptor (CCR2) signaling,^3^ and begins to differentiate into macrophages, which replaced the resident macrophages in the ischemic area within 24 hours post-MI.^4^ Macrophages are highly plastic and can adopt various activation states.^5,6^ At 1 to 3 days post-MI, monocyte-derived macrophages exhibit pro-inflammatory characteristics, secreting inflammatory cytokines and chemokines. On the one hand, they contribute to the clearance of necrotic cells, facilitating the repair of infarcted heart. On the other hand, excessive inflammatory reaction may extend ischemic injury by damaging surviving cardiomyocytes of the border zone and promoting protease activity.^7^ Increased interleukin 6 are associated with poor outcomes in MI patients.^8^ Pro-inflammatory macrophages impair healing^9^; whereas suppressing pro-inflammatory macrophage polarization reduces necrosis of cardiomyocytes^10^ and cardiac rupture^11^ post-MI. Targeting CCR2 using siRNA decreases monocyte infiltration, leading to a decrease in infarct size and improvement of cardiac function.^12,13^

The extracellular matrix (ECM) which is a dynamic structure is critical in cardiac injury, repair, and remodeling post-MI. Macrophages and inflammation also regulate ECM. Infiltrated macrophage is one of the primary sources of metalloproteinases (MMPs), which mediate the degradation of ECM.^5^ ECM degradation facilitates cardiac healing in the early stages. Optimal scar formation requires proper synthesis, cross-linking, and ECM alignment, which helps to maintain cardiac function post-MI. However, excessive ECM degradation may result in insufficient collagen deposition, leading to left ventricle (LV) thinning and dilation. Additionally, plasma MMP9 levels are correlated with LV dysfunction post-MI.^14^ Conversely, knockout (KO) of *Mmp9* or selective MMP inhibition can decrease MI-induced cardiac rupture and LV dilation in mice.^15,16^ Moreover, overexpression of tissue inhibitor of metalloproteinase 1 (*Timp1*) preserves cardiac function in MI rats;^17^ whereas, *Timp1* KO exacerbates LV remodeling post-MI.^18^ Thus, modulation of the macrophage function to reduce MMP expression or activation may decrease the risk of post-MI cardiac injury.

Pattern recognition receptors (PRRs) recognize DAMPs and induce activation of downstream inflammatory pathways. The proteins of the intracellular caspase recruitment domain (CARD) family act as linkers between PPRs and downstream signaling molecules.^19–21^ In particular, CARD9 is a critical adapter of activated PRRs, such as C-type lectin receptors (CLRs), intracellular nucleotide oligomerization domain (NOD)-like receptors, toll-like receptors (TLRs), and some other intracellular nucleic acid-sensing PPRs in response to fungal, bacterial, viral, and parasitic infections.^22–26^ CARD9 is mainly expressed by myeloid cells. With microorganism stimulation, PPRs recruit spleen tyrosine kinase (Syk) or receptor interacting protein 2 (RIP2), which further activates CARD9. After activation, CARD9 binds to the CARD domain of BCL10 immune signaling adaptor, which interacts with MALT1 paracaspase to form a CARD9/BCL10/MALT1complex, activating downstream nuclear factor kappa-light-chain-enhancer of activated B cells (NF-κB) and mitogen-activated protein kinases (MAPKs) signaling.^23,27,28^ Mice with *Card9* knockout (KO) are susceptible to certain microbial infections.^28,29^ CARD9 also participates in noninfectious inflammation regulation.^30–32^ Furthermore, *Card9* KO improves insulin resistance by blocking MAPK pathways in high-fat diet treated mice,^31^ and knockdown of *Card9* in acute pancreatitis rats inhibits NF-κB and p38 MAPK signaling and attenuates pancreatic, lung, and liver tissue injury.^32^ However, whether CARD9 is a key regulator between innate immunity and cardiac injury in MI remains unclear. In a previous study, we showed that CARD9 mediated necrotic cell-induced NF-κB signaling activation in macrophages and promoted inflammatory cytokine expression and neointima formation in vein grafts.^33^ The role of CARD9 has also been studied in several cardiac disease models. *Card9* depletion decreases angiotensin II-induced cardiac fibrosis,^34^ prevents pressure overload-induced heart failure and fibrosis,^35^ and alleviates cardiac injury in obese mice.^36^ Exploring the function of CARD9 and underlying mechanisms in cardiac injury and remodeling post-MI will provide us the new targets or strategies for MI therapy.

In the present study, we investigated the expression of CARD9 in ischemic myocardium and revealed its role in cardiac injury and remodeling post-MI using *Card9* KO mice. We found that CARD9 mainly located in macrophages and its regulation of lipocalin 2 (LCN2) through NF-κB activation may contribute to its adverse effects post-MI.

## Results

### CARD9 expression is increased in infarcted heart

**Fig. 1:**
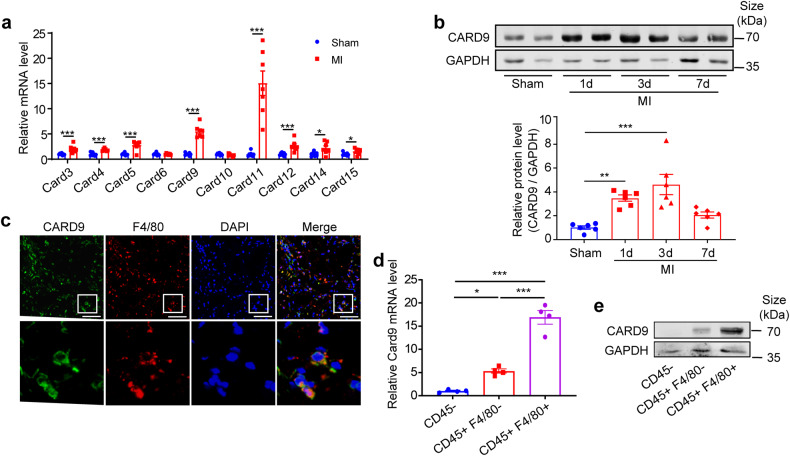
CARD9 is increased in mouse hearts post-MI and mainly expressed in macrophages. **a** The mRNA levels of *Card* family genes in below-ligature cardiac tissues at 3 days post-MI, quantified as fold induction with respect to the sham group after normalization to *Gapdh* (*n* = 7). **b** CARD9 protein levels in cardiac tissues of sham and MI model mice at the indicated time points post-MI, quantified as fold induction with respect to the sham group after normalization to GAPDH (*n* = 6). **c** Representative co-staining of CARD9 and F4/80 on cardiac sections at 3 days post-MI (*n* = 4). Nuclei were stained with DAPI. CD45^-^, CD45^+^ F4/80^-^ and CD45^+^ F4/80^+^ cells sorted from hearts of mice at 3 days post-MI. **d** *Card9* mRNA expression in the three population of MACS-sorted cells, quantified as fold change with respect to CD45^-^ group after normalization to *Gapdh*. Hearts from 4-5 mice were pooled as one sample; n = 4 samples. **e** CARD9 protein levels in three population of FACS-sorted cells. Hearts from three mice were used for sorting. Scale bar: 50 µm. Data represent the mean ± SEM. Two-tailed unpaired *t*-test (a) and one-way ANOVA followed by Tukey’s test for post hoc comparison (b and d) were used for statistical analysis. *p < 0.05, **p < 0.01, ***p < 0.001

MI was induced in mice. Three days after infarction, the expression of *Card* family genes was examined, and the mRNA levels of *Card9* and *Card11* were found to be increased the most among all the *Card* family genes (Fig. 1a). Subsequently, we assayed the protein levels of CARD9 in myocardium at different time following infarction and found that CARD9 expression was induced as early as 1 day, peaked on 3 days, and descended on 7 days post-MI (Fig. 1b). To determine the cellular localization of CARD9, immunofluorescent staining was performed on infarcted heart sections. We observed that CARD9 co-stained with F4/80, a macrophage marker, indicating that CARD9 was expressed in macrophages (Fig. 1c). To explore CARD9 expression levels in various heart cells, we isolated CD45^-^, CD45^+^ F4/80^-^, and CD45^+^ F4/80^+^ cells from infarcted mouse hearts using enzyme digestion and magnetic- or fluorescence-activated cell sorting. We found that *Card9* mRNA expression was highest in CD45^+^ F4/80^+^ macrophages, lower in CD45^+^ F4/80^-^ bone marrow (BM)-derived cells, and lowest in CD45^-^ cells (Fig. 1d). Furthermore, a large amount of CARD9 protein was observed in CD45^+^ F4/80^+^ macrophages, a small amount of CARD9 in CD45^+^ F4/80^-^ cells, and virtually no expression of CARD9 in CD45^-^ cells (Fig. 1e), which was consistent with the mRNA data. Because CARD9 expression was mainly detected in CD45^+^ cells, we investigated CARD9 expression in distinct populations of CD45^+^ immune cells. We obtained a published single-cell RNA sequencing (scRNA-seq) dataset (GSE163465)^37^ of cardiac CD45^+^ cells in mice after MI from the Gene Expression Omnibus (GEO) database and observed that macrophages contributed the most to *Card9* expression post-MI (Supplementary Fig. 1). These results confirmed *Card9* expression in macrophages post-MI.

### *Card9* KO enhances LV function and attenuates reversed remodeling post-MI

**Fig. 2:**
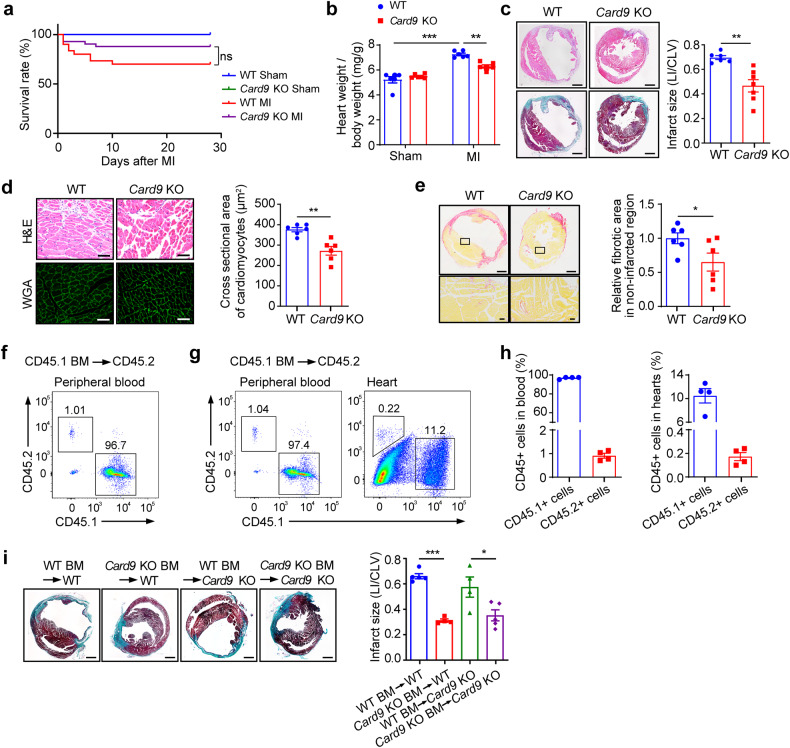
*Card9* knockout attenuates infarct size and adverse left ventricular remodeling post-MI. **a** Survival analysis of mice post-MI (*n* = 8, 8, 30, 41). **b** Ratios of heart weight to body weight at 28 days post-MI (*n* = 6). **c** H&E and Masson’s trichrome staining of cardiac sections at 28 days after MI. Infarct size is presented as the ratio of infarcted area length to left ventricular circumference (*n* = 6). **d** H&E and wheat germ agglutinin (WGA) staining of non-infarcted area. The bar graft shows cross-sectional area of myocytes (*n* = 6). **e** Sirus Red staining. Fibrotic area in non-infarcted region was measured and quantified as fold induction with respect to WT group (*n* = 6). **f** Flow cytometric analysis of CD45.1^+^ and CD45.2^+^ cells in the peripheral blood of CD45.2 mice transplanted with BM cells from CD45.1 mice (*n* = 3). **g** Detection of donor BM cell infiltration into the heart at 3 days post-MI. **h** Bar grafts show the percentage of CD45.1^+^ or CD45.2^+^ cells in g (*n* = 4). **i** Masson’s trichrome staining on cardiac sections from mice with BMT at 28 days post-MI and measurement of infarct size (*n* = 4-5). Scale bar: 1 mm (c, e top and i), 50 μm (d and e bottom). Data represent the mean ± SEM. One-way ANOVA followed by Tukey’s test (b and i) and two-tailed unpaired *t*-test (c, d, and e) were used for statistical analysis. *p < 0.05, **p < 0.01, ***p < 0.001

To determine the involvement of CARD9 in MI, we used *Card9* KO mice. The survival of the mice was analyzed and cardiac function was determined at 28 days post-MI. *Card9* KO increased the survival rate of MI mice from 70% to 82% (Fig. 2a), significantly decreased the LV end-systolic internal diameter, and increased the LV ejection fraction (Supplementary Table 1). Collectively, our results demonstrated that *Card9* deficiency restores cardiac function post-MI.

We further investigated whether CARD9 affects pathological remodeling. *Card9* KO attenuated heart weight/body weight ratio (Fig. 2b). Moreover, *Card9* KO reduced the infarct size compared with wild-type (WT) mice at 28 days post-MI (Fig. 2c) and suppressed the enlargement of cardiomyocytes (Fig. 2d) and interstitial fibrosis in non-ischemic regions (Fig. 2e). Collectively, these data provide the first evidence that *Card9* KO attenuates adverse cardiac remodeling following MI.

To exclude the influence of *Card9* KO on other *Card* family member expression, we examined the mRNA levels of these genes in BM-derived macrophages (BMDMs), BM neutrophils, heart, liver, lung, and kidney tissues, and found *Card9* KO did not affect *Card* family expression in these cells and tissues (Supplementary Fig. 2). Moreover, *Card9* KO mice exhibited no changes in body weight (Supplementary Fig. 3a) and the morphology of vital organs, such as the liver, lungs, and kidneys (Supplementary Fig. 3b).

As the expression of *Card11* was also significantly increased post-MI, we evaluated its role. We found that *Card11* KO did not change infarct size or cardiac function at 28 days post-MI (Supplementary Fig. 4a, b).

### *Card9* KO in BM cells results in protective effects post-MI

In the early post-MI stages, most macrophages are derived from infiltrated monocytes,^4,38,39^ exhibiting pro-inflammatory and pro-injury effects. CCR2 is a commonly used marker to distinguish the origin of cardiac macrophages. CCR2^-^ macrophages are embryonically derived, and CCR2^+^ macrophages are derived from circulating monocytes.^4,40^ We analyzed *Card9* expression in CCR2^+^ and CCR2^-^ macrophages using the cardiac CD45^+^ scRNA-seq dataset (GSE163465)^37^ and found the expression of *Card9* seemed marginally higher in CCR2^+^ macrophages, although the difference was not significant (*p* = 0.0556, Supplementary Fig. 5). To further explore the importance of CARD9 expression in infiltrating myeloid cells in MI, BM transplantation (BMT) experiments were performed in WT and *Card9* KO mice. We used mice expressing the CD45.1 allele as donors and CD45.2 mice with the same background as recipients to estimate BM reconstitution. Flow cytometric analysis confirmed that over 95% of the blood cells (with erythrocytes removed) from the CD45.2 recipients expressed the CD45.1 allele at 8 weeks after BMT (Fig. 2f), indicating successful BM reconstitution. These CD45.2 recipient mice were subjected to MI, and we observed that more than 10% of cells in the heart expressed CD45.1, whereas less than 0.5% of cells were CD45.2^+^ at 3 days post-MI (Fig. 2g, h), suggesting that many donor BM-derived cells had infiltrated into the heart.

WT and *Card9* KO recipient mice were induced MI after BM reconstitution. We found that transplantation of *Card9* KO BM cells into WT mice reduced the infarct size at 28 days post-MI compared to that in WT mice transplanted with WT BM cells. In contrast, transplantation of WT BM cells into *Card9* KO mice increased infarct size compared to that in *Card9* KO mice transplanted with *Card9* KO BM cells (Fig. 2i). These results confirmed that CARD9 from BM-derived cells contributed to cardiac injury and remodeling post-MI.

### *Card9* KO has no significant effect on angiogenesis but inhibits cardiomyocyte apoptosis and MMP expression

To explore the mechanism for CARD9 regulating MI, we first evaluated the angiogenic effect of *Card9* KO by staining endothelial cell marker at 7 days post-MI and observed that *Card9* KO did not significantly increase the number of microvessels in the border regions (Supplementary Fig. 6a). Moreover, we isolated BM-derived macrophages from WT and *Card9* KO mice (Supplementary Fig. 6b) and treated them with necrotic cells. We found that the levels of vascular endothelial growth factor A and fibroblast growth factor 2 were lower in *Card9*-deficient cells (Supplementary Fig. 6c), suggesting that mechanisms other than angiogenesis are responsible for the protective role of *Card9* KO.

**Fig. 3:**
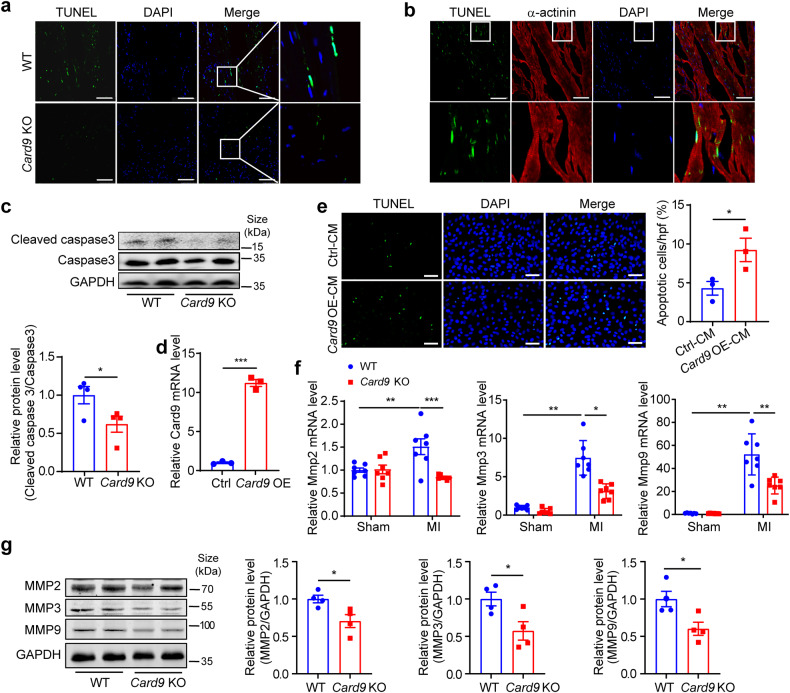
*Card9* knockout attenuates apoptosis of cardiomyocytes and *Mmp* expression post-MI. **a** TUNEL assay in border region of hearts at 3 days after MI. **b** TUNEL and α-actinin co-staining in the border region of WT hearts. Nuclei were stained with DAPI. **c** Cleaved caspase 3 protein levels in WT and *Card9* KO hearts at 3 days after MI, quantified as fold induction with respect to WT after normalization to Caspase 3 (*n* = 4). **d** qPCR analysis of *Card9* expression in *Card9*-overexpressing (OE) and Ctrl RAW264.7 cells, quantified as fold change with respect to Ctrl after normalizing to *Gapdh* (*n* = 3). **e** TUNEL assay in H9C2 cells treated with conditioned medium from Ctrl and *Card9* OE RAW264.7 cells. The percentage of apoptotic cells was calculated (*n* = 3). **f** The mRNA expression of *Mmp2*, *Mmp3* and *Mmp9* in hearts at 3 days after MI (*n* = 7), quantified as fold induction with respect to WT Sham after normalization to *Gapdh*. **g** MMP2, MMP3 and MMP9 protein levels in hearts, quantified as fold change with respect to WT after normalization to GAPDH (*n* = 4). Scale bar: 50 μm. Data represent the mean ± SEM. Two-tailed unpaired *t*-test (c, d, e and g) and one-way ANOVA followed by Tukey’s test (f) were used for statistical analysis. *p < 0.05, **p < 0.01

Subsequently, we examined immune cell infiltration and found reduced macrophage and neutrophil recruitment in the border region of *Card9* KO mice at 3 days post-MI (Supplementary Fig. 7a, b) and fewer apoptotic cells (Fig. 3a), which were identified as cardiomyocytes (Fig. 3b). Moreover, cleaved caspase 3 in *Card9* KO hearts were reduced (Fig. 3c). Thus, *Card9* KO protects cardiomyocytes from secondary apoptosis after MI.

To further evaluate the function of macrophage *Card9* on cardiomyocyte apoptosis, *Card9* was overexpressed in RAW264.7 macrophages (Fig. 3d). H9C2 cardiomyocytes were treated with conditioned medium from *Card9*-overexpressing (OE) and control RAW264.7 cells, and it was observed that *Card9* OE conditioned medium increased the apoptosis of H9C2 cells (Fig. 3e).

Excessive expression of ECM-degrading proteases can cause cardiac injury following MI. We then assessed the expression of *Mmp* genes and found that *Card9* KO decreased *Mmp2*, *Mmp3*, and *Mmp9* mRNA levels (Fig. 3f) as well as the protein levels (Fig. 3g). Additionally, MMP2 and MMP9 staining were reduced in *Card9* KO hearts (Supplementary Fig. 7c, d). Regulation of MMPs may contribute to the protective effects in *Card9* KO mice post-MI. Moreover, we examined ECM-related gene expression 7 days post-MI and found that *Card9* KO increased *Col1a1*, *Col3a1*, and *Fn1* mRNA expression (Supplementary Fig. 8).

### *Card9* KO attenuates LCN2 expression in macrophages via NF-κB post-MI

**Fig. 4:**
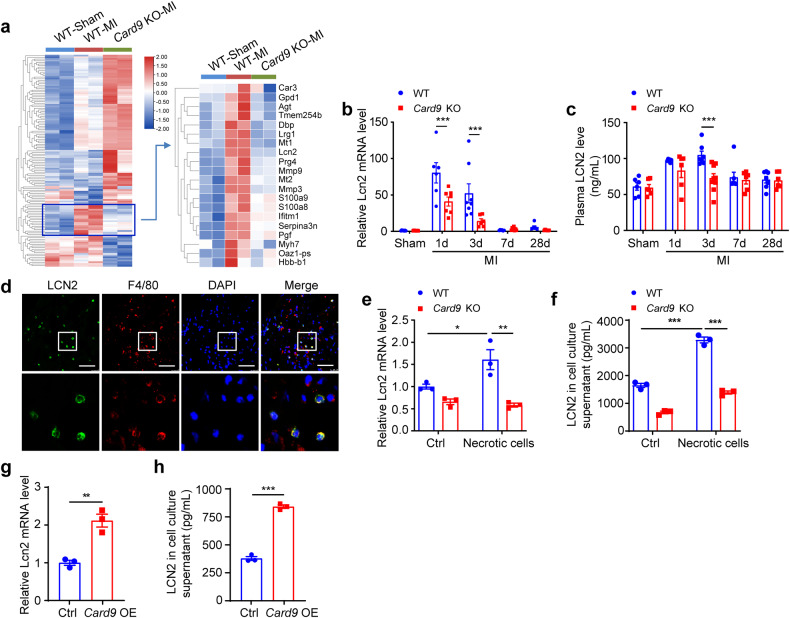
*Card9* knockout attenuates *Lcn2* expression in macrophages post-MI. **a** Heat map of DEGs between WT-MI and *Card9* KO-MI groups (*n* = 2). **b** *Lcn2* mRNA expression in WT and *Card9* KO hearts post-MI (*n* = 6–8). **c** Detection of LCN2 plasma levels by ELISA (*n* = 6–9). **d** Co-staining of LCN2 and F4/80 in the border region of WT cardiac tissues at 3 days after MI. Nuclei were stained with DAPI. **e** *Lcn2* mRNA expression in necrotic cell-stimulated WT and *Card9* KO BMDMs, quantified as fold induction with respect to WT Ctrl after normalization to *Gapdh*. **f** ELISA analysis of LCN2 levels in cell culture supernatant (*n* = 3). **g** qPCR analysis of *Lcn2* expression in *Card9* OE RAW264.7 cells, quantified as fold change with respect to Ctrl after normalizing to *Gapdh*. **h** ELISA analysis of LCN2 levels in cell culture supernatant (*n* = 3). Scale bar: 50 μm. Data represent the mean ± SEM. Two-way ANOVA followed by Bonferroni’s test (**b**, **c**), one-way ANOVA followed by Tukey’s test (e and f), and two-tailed unpaired *t*-test (g and h) were used for statistical analysis. *p < 0.05, **p < 0.01, ***p < 0.001

To investigate the molecular mechanism of CARD9 regulating the extent of cardiac injury after MI, we conducted RNA sequencing using infarcted heart tissue from WT and *Card9* KO mice. Gene ontology (GO) enrichment analysis on differentially expressed genes (DEGs) between WT MI and *Card9* KO MI groups showed that the downregulated genes were associated with secreted proteins, proteins involved in inflammation, and MMP-related proteins; whereas, the upregulated genes were ECM remodeling-related (Supplementary Fig. 9a, b). Among the downregulated genes in *Card9* KO hearts, the change in *Lcn2* expression compared to that in WT hearts was the most significant (Fig. 4a and supplementary Fig. 9c). In contrast, its expression was increased in MI hearts compared to that in sham hearts. The regulation of *Lcn2* mRNA expression by CARD9 was confirmed in the heart post-MI (Fig. 4b). Moreover, we found the mRNA levels of *Lcn2* were elevated at 1 day and 3 days after MI and returned to the baseline level at 7 days (Fig. 4b). The change in plasma LCN2 levels also showed a similar pattern (Fig. 4c); however, *Card9* KO inhibited the increase in LCN2 levels at 3 days post-MI (Fig. 4b, c). These results suggest that LCN2 may be produced by inflammatory cells. Therefore, we co-stained LCN2 with macrophage marker F4/80 and found that LCN2 was expressed in macrophages (Fig. 4d). Subsequently, we stimulated BMDMs from WT and *Card9* KO mice with necrotic cells, and found that *Card9* KO counteracted the increase in *Lcn2* mRNA expression (Fig. 4e) and protein release into the cell culture supernatant (Fig. 4f). In *Card9* OE RAW264.7 cells, the mRNA expression (Fig. 4g) and protein release (Fig. 4h) of LCN2 also increased.

**Fig. 5:**
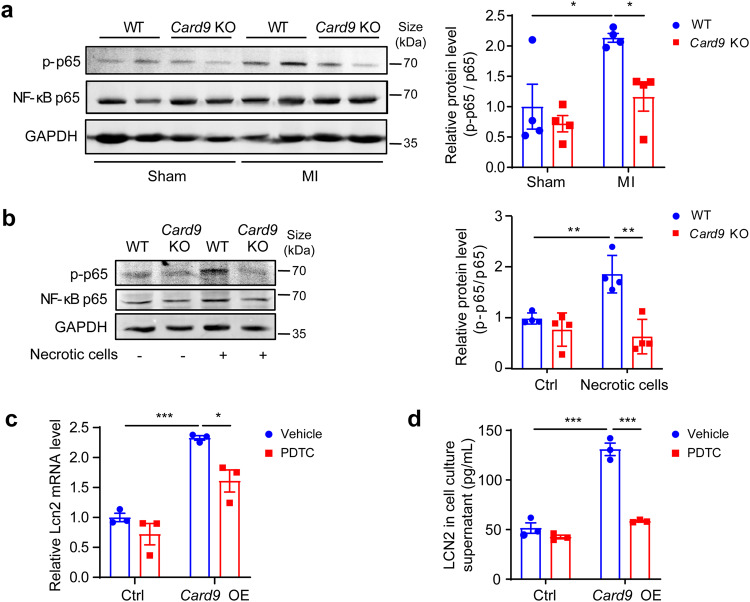
CARD9 regulates *Lcn2* expression via NF-κB activation. **a** Protein levels of phosphorylated p65 in WT and *Card9* KO hearts at 3 days after MI, quantified as fold induction with respect to WT Sham after normalization to total p65 (*n* = 4). **b** Protein level of phosphorylated p65 in necrotic cell-treated WT and *Card9* KO macrophages, quantified as fold induction with respect to WT Ctrl after normalization to total p65 (*n* = 4). *Card9* OE RAW264.7 cells were treated with ammonium pyrrolidine dithiocarbamate (PDTC). **c** *Lcn2* mRNA expression was analyzed by qPCR, quantified as fold change with respect to Ctrl vehicle after normalizing to *Gapdh* (*n* = 3). **d** LCN2 protein in cell culture supernatant detected by ELISA (*n* = 3). Data represent the mean ± SEM. One-way ANOVA followed by Tukey’s test were used for statistical analysis. *p < 0.05, **p < 0.01, ***p < 0.001

LCN2 is reported to be regulated by NF-κB,^41,42^ and we have previously observed that *Card9* KO attenuates necrotic smooth muscle cells-induced NF-κB activation.^33^ Thus, we hypothesized that CARD9 regulated LCN2 via NF-κB. To test this hypothesis, we first examined NF-κB activation in cardiac tissue after MI, and found that *Card9* KO attenuated the phosphorylation of its p65 subunit (Fig. 5a). *Card9* KO also abolished necrotic cell-induced phosphorylation of p65 in BMDMs (Fig. 5b). When *Card9* OE RAW264.7 macrophages were treated with NF-κB inhibitor, the mRNA expression and protein release of LCN2 were inhibited (Fig. 5c, d). These data indicated that CARD9 may regulate LCN2 expression through NF-κB.

### LCN2 mediates the effect of CARD9 post-MI

**Fig. 6:**
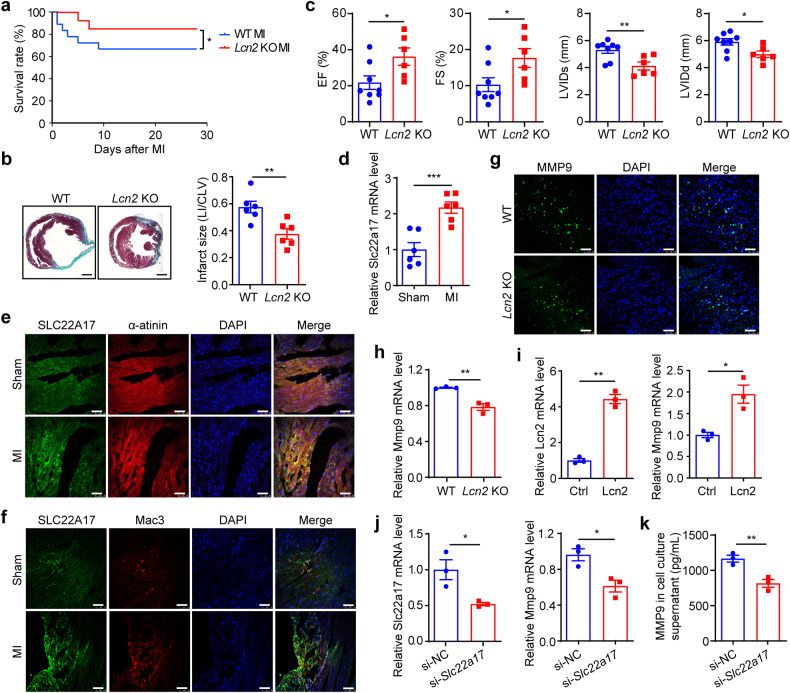
LCN2 mediates cardiac injury in MI and enhanced MMP9 expression in macrophages. **a** Survival curve of WT and *Lcn2* KO mice post-MI (*n* = 18,13). **b** Masson’s trichrome staining at 28 days post-MI. Infarct size is presented as the ratio of infarcted area length to left ventricular circumference (*n* = 6). **c** Echocardiographic analysis of left ventricular end-systolic internal diameter (LVIDs), left ventricular end-diastolic internal diameter (LVIDd), ejection fraction (EF) and fractional shortening (FS) (*n* = 8, 6). **d** *Slc22a17* mRNA expression in cardiac tissues of sham and 3 days post-MI groups, quantified as fold induction with respect to Sham after normalization to *Gapdh* (*n* = 6). Co-staining of SLC22A17 and Mac 3 (**e**) or α-actinin (**f**) in the border region of WT hearts from sham and 3 days post-MI mice. **g** MMP9 staining in WT and *Lcn2* KO mouse hearts at 3 days post-MI. Nuclei were stained with DAPI. **h** *Mmp9* mRNA expression in BMDMs from WT and *Lcn2* KO mice at 3 days post-MI, quantified as fold induction with respect to WT after normalization to *Gapdh* (*n* = 3). **i** qPCR analysis of *Lcn2* and *Mmp9* expression in *Lcn2*-overexpressed RAW264.7 cells, quantified as fold induction with respect to Ctrl after normalizing to *Gapdh* (*n* = 3). **j** BMDMs were transfected with Slc22a17 siRNA and Slc22a17 and Mmp9 expression was assayed by qPCR, quantified as fold induction with respect to si-negative ctrl (NC) after normalization to *Gapdh* (*n* = 3). **k** ELISA analysis of MMP9 protein in cell culture supernatant (*n* = 3). Scale bar: 1 mm (**b**) and 50 μm (**e**, **f** and **g**). Data represent the mean ± SEM. Two-tailed unpaired *t*-test was used for statistical analysis. *p < 0.05, **p < 0.01, ***p < 0.001

To determine the function of LCN2 in MI, we ligated the left coronary artery of *Lcn2* KO mice. We observed that *Lcn2* KO considerably increased the survival (Fig. 6a), diminished infarct size (Fig. 6b), and ameliorated cardiac function (Fig. 6c) post-MI in these mice. However, the administration of recombinant mouse LCN2 to *Card9* KO mice after MI increased death, although it was not statistically significant owing to the limited number of mice (Supplementary Fig. 10a), enlarged infarct size (Supplementary Fig. 10b), and worsened cardiac function (Supplementary Fig. 10c).

### LCN2 induces *Mmp9* expression in macrophages

To explore the mechanism by which LCN2 regulates the extent of cardiac injury after MI, we first identified the expression and cellular localization of the LCN2 receptor solute carrier family 22 member 17 (SLC22A17) in the cardiac tissue. We found *Slc22a17* mRNA expression elevated after MI (Fig. 6d), and immunofluorescence staining revealed SLC22A17 expression in both cardiomyocytes and macrophages (Supplementary Fig. 11a, b). Moreover, the intensity of SLC22A17 staining was enhanced in the MI hearts (Fig. 6e, f).

LCN2 stimulation induces cardiomyocyte apoptosis,^43^ possibly contributing to the effect of LCN2 on cardiac injury post-MI. Additionally, we assessed MMP9 expression in MI hearts and observed less MMP9 staining in *Lcn2* KO cardiac sections (Fig. 6g). We isolated BMDMs from WT and *Lcn2* KO MI mice and detected decreased *Mmp9* expression in *Lcn2* KO cells (Fig. 6h). Furthermore, *Lcn2*-overexpression in RAW264.7 resulted in increased *Mmp9* expression (Fig. 6i). These results suggested that LCN2 regulates MMP9 expression after MI. Subsequently, we interrupted SLC22A17 expression in WT BMDMs to identify the involvement of SLC22A17 in LCN2-mediated regulation of MMP9 and found that SLC22A17 knockdown reduced Mmp9 mRNA expression (Fig. 6j) and MMP9 release into the supernatant with recombinant LCN2 treatment (Fig. 6k).

## Discussion

Our study revealed a critical role for CARD9 in macrophage in regulating cardiac injury post-MI. CARD9 is an adapter molecule that mediates pathogen-associated molecular pattern (PAMP)-activated downstream signaling of PRRs. However, whether CARD9 is involved in DAMP-induced innate immunity remains unclear. Our previous study showed that necrotic smooth muscle cells in vein grafts activated NF-κB in macrophages through CARD9, revealing the function of CARD9 in mediating necrotic cell-induced inflammation for the first time.^33^ MI also activates innate immunity. DAMPs released by necrotic cells activate inflammatory cells that infiltrate the border region. Dectin-1 and Dectin-2, which belong to the CLR family of PRRs, are involved in cardiac ischemia.^44,45^ We detected an increase in *Card9* expression in the myocardium, beginning as early as 1 day after MI, peaking at 3 days, and decreasing thereafter, suggesting that CARD9 is involved in myocardial ischemic injury. Furthermore, using *Card9* KO mice, we identified CARD9 as a key mediator of injury and adverse remodeling after MI.

Our results demonstrated that CARD9 was mainly expressed in CD45^+^ F4/80^+^ macrophages. Furthermore, a BMT experiment confirmed that CARD9 in BM-derived cells mediated myocardial ischemic injury. Alteration of the ECM is vital for the repair post-MI, particularly for collagen-based scar formation. Macrophages, particularly classically activated macrophages, are an essential source of MMPs that degrade ECM following MI.^46^ Strategies that alter macrophage polarization and decrease MMP expression or activation may help to preserve the structural integrity and function of the heart post-MI. We found that *Card9* KO attenuated *Mmp9* expression following MI, which may have contributed to the protective effect of *Card9* KO. In exploring the downstream consequences of CARD9 activation, we identified LCN2 using RNA sequencing. LCN2 is a glycoprotein first identified in neutrophils. However, further studies have also found that LCN2 may be released by various cells, such as epithelial cells, macrophages, and hepatocytes, during inflammation or injury.^47–49^ LCN2 binds directly to MMP9, thereby protecting it from degradation and enhancing its proteolytic activity.^50^ LCN2 serum levels are increased in both patients with MI and animal models^48,51^ and are associated with adverse outcomes.^51,52^ Here, we demonstrated that *Lcn2* deletion enhanced cardiac function and limited infarct scar size post-MI, which is consistent with a previous study.^53^ The direct interaction between LCN2 and MMP9 may be one of the mechanisms underlying the post-MI regulatory role of LCN2. Moreover, LCN2 has been reported to induce apoptosis in cultured cardiomyocytes by inhibiting autophagy and increasing intracellular iron accumulation,^43^ and LCN2-siderophore-iron complexes increase ROS generation and decrease oxidative phosphorylation in mitochondria, which can also cause injury to cardiomyocytes.^54^ We found that *Lcn2* overexpression increased *Mmp9* expression in macrophages, and *Lcn2* KO reduced MMP9 post-MI. Thus, LCN2 may mediate post-MI cardiac injury by regulating cardiomyocyte apoptosis and MMP9 expression.

CARD9 is located in the cytoplasm and, therefore, cannot directly regulate the expression of *LCN2*. However, the presence of an NF-κB response element in the *LCN2* promoter has been reported, and NF-κB is a positive regulator of *LCN2* expression.^41,42,55^ Our study showed that CARD9 mediated NF-κB activation in the heart following MI. When NF-κB activation was inhibited, *Card9* overexpression-induced *Lcn2* expression in macrophages was diminished, which is consistent with the results of *Card9* KO. Our results suggest that CARD9 might regulate *Lcn2* expression in macrophages via NF-κB activation.

CARD9 also expresses in neutrophils and dendritic cells in addition to macrophages. CARD9 deficiency impairs neutrophil function in fungal infections and autoimmune diseases.^30,56,57^ Neutrophil-specific deletion of CARD9 inhibits inflammatory reactions in mice with autoantibody-induced arthritis and dermatitis.^30^ Neutrophils infiltrate immediately after the onset of MI and are involved in injury and repair processes. We detected much lower CARD9 expression in CD45^+^ F4/80^-^ cells than in macrophages, indicating that CARD9 mainly regulates macrophage function; however, we cannot exclude the effect of CARD9 on other myeloid cells. CARD9 has been reported to be increased in H9C2 cells and neonatal rat cardiomyocytes treated with hypoxia/reoxygenation or hydrogen peroxide, inhibiting apoptosis and promoting autophagy of cardiomyocytes during myocardial ischemia/reperfusion (I/R).^58,59^ The differences with our findings may be attributed to the distinct mechanisms of I/R and MI. Indeed, in I/R, oxidative stress owing to consistent ROS production during reperfusion is the primary cause of cardiomyocyte injury; whereas in MI, ischemia induces necrosis and apoptosis of cardiomyocytes, and infiltration of immune cells in the border region contributes to healing or further injury.

Revealing the role of CARD9 and LCN2 in MI provides us potential molecular targets for cardiac ischemic diseases. Because LCN2 is a secreted protein, blocking its effect using antibodies may be a new therapeutic strategy for MI. Several other *Card* genes have been detected increased post-MI in addition to *Card9*. A previous study shows that *Card3* KO in mice alleviates LV remodeling and dysfunction post-MI and *Card3* overexpression in cardiomyocytes has the opposite effects.^60^ CARD6 and CARD15 protect against pressure overload-induced cardiac hypertrophy.^61,62^ The effects of other CARD family members in cardiac ischemia can be explored in the future.

In conclusion, the present study demonstrated the vital role of the innate immune protein CARD9 in cardiac injury after MI. CARD9 upregulated *Lcn2* expression via NF-κB activation in macrophages, which in turn increased MMP9 expression, contributing to the enlargement of the injured area and adverse remodeling (Supplementary Fig. 12).

## Materials and methods

### Animals and MI model

*Card9* KO mice were constructed as previously described.^28^ *Lcn2* KO mice were obtained from the Jackson Laboratory (Bar Harbor, ME, USA). CD45.1 strain mice were obtained from Cyagen (Suzhou, Jiangsu, China). The above strains are all on a C57BL/6 genetic background. Ten- to fourteen-week -old *Card9* or *Lcn2* KO male mice were used, and Wild-type (WT) littermates and C57BL/6 mice were used as controls. All mice could freely get food and water. The experiments were conformed to the US National Institutes of Health Guide for the Care and Use of Laboratory Animals and approved by the Institutional Animal Care and Use Committee of Capital Medical University. Experimental MI in mice was induced by ligation of the left anterior descending coronary artery as previously described.^63^ Anesthesia of mice was achieved with 1.5- 3% isoflurane in 100% oxygen. The same surgery was performed on sham group mice without ligation. Recombinant mouse LCN2 (Sino Biological, Beijing, China) was given to *Card9* KO mice via angular vein injection on day -0, -1, and -3 post-MI at a dose of 30 µg per mouse. Mice were sacrificed by carbon dioxide (CO_2_) at different time points post-MI, and their hearts were harvested for further experiments.

### Cell isolation and culture

BMDMs were isolated following protocols we previously described.^33^ Obtained cells were cultured in RPMI-1640 medium (Gibco, Waltham, MA, USA) supplemented with 10% fetal bovine serum (FBS, Gibco), penicillin-streptomycin and 50 ng/mL of macrophage colony-stimulating factor (PeproTech, Rocky Hill, NJ). Three days later, the BMDMs were harvested with trypsin and reseeded in culture plates for further use.

The RAW264.7 cells and H9C2 cardiomyocytes were maintained in Dulbecco’s modified Eagle’s medium (DMEM; Gibco) with 10% FBS and penicillin-streptomycin. Confluent monolayers of H9C2 cells were harvested by trypsinization and resuspended in the BMDM growth medium at the density of 1×10^6^ cells/mL. Necrotic cardiomyocytes used for macrophage stimulation in vitro were produced by subjecting the cell suspensions to five freeze - thaw cycles in liquid nitrogen and 37 °C.^64,65^

Necrotic cells were given to WT and *Card9* KO BMDMs at a 3:1 ratio (necrotic cells: macrophages), and an equal volume of medium with five freeze - thaw cycles was used for control experiments. Four hours after stimulation, the medium was removed and fresh medium was added. After an additional 20 h of culture, the supernatants of cells were collected for LCN2 detection.

### Bone marrow transplantation

BMT was carried out as previously described.^66^ Briefly, eight- to ten-week-old male recipient mice received total body irradiation at a dose of 10 Gy from a cobalt-60 source. BM cells were isolated from age-matched male donors and suspended in a serum-free RPMI-1640 medium. Four hours after irradiation, a 200 µL aliquot of BM cell suspension containing 1 × 10^7^ cells was injected into recipient mice through the angular vein. The mice were kept with acidified water containing 100 mg/L fluconazole and 100 mg/L levofloxacin after irradiation for 2 weeks and switched to acidified water without antibiotics for an additional 6 weeks. Eight weeks after BMT, the mice were given a MI surgery. CD45.2 strain mice received BM cells from CD45.1 strain were used for BM reconstitution evaluation. Cells with CD45.1 or CD45.2 expression in the blood were analyzed by flow cytometry.

### Echocardiographic imaging

Transthoracic echocardiography of mice was carried out with a Vevo 770 system^66^ (Fujifilm VisualSonics, Ontario, Canada) during anesthesia with 1.5–3% isoflurane in 100% oxygen at 28 days post-MI. Two-dimensional images of the short axis of LV were taken. LV wall thickness and internal diameters were measured for five consecutive beats and averaged in M-mode. Ejection fraction and fractional shortening were calculated.

### Gene overexpression and knockdown

A plasmid expressing mouse *Card9* with a C-terminal FLAG-tag and a neomycin resistance gene was constructed by GeneChem (Shanghai, China). A plasmid expressing mouse *Lcn2* with a C-terminal FLAG-tag was purchased from Sino Biological. siRNA against mouse *Slc22a17* (target sequence: 5’-CCATCACAACCTTCTCTGT-3’) was synthesized by Rui Biotech (Guangzhou, China).

For *Card9* overexpression, *Card9* plasmid transfection into RAW264.7 cells were carried out using Lipofectamine 3000 (Invitrogen), and the same vector expressing GFP was used as a control. The culture medium was discarded and a fresh selective medium containing G418 (Geneticin, Gibco) was added to cells after 24 hours. The selective medium was refreshed every 3 days for a period of 14 days, after which *Card9*-overexpressing cells were obtained. Equivalent numbers of *Card9*-overexpressing and control RAW264.7 cells were plated, and the cell culture supernatant was collected after 48 hours for detection of LCN2 levels and treatment of H9C2 cells as conditioned medium. Cells were treated with ammonium pyrrolidinedithiocarbamate (PDTC; Sigma-Aldrich, St. Louis, MO, USA), the NF-κB inhibitor, at 10 μM for 24 hours, and then RNA was isolated and the supernatant was collected for LCN2 detection. The same volume of vehicle was used as control.

For *Lcn2* overexpression, RAW264.7 cells were transfected with *Lcn2* plasmid using the 4D-Nucleofector X-unit (Lonza, Basel, Switzerland). An empty plasmid was used as a control. Forty-eight hours after transfection, RNA was isolated and overexpression of *Lcn2* was confirmed by quantitative PCR (qPCR) analysis.

For Slc22a17 knockdown, WT BMDMs were transfected with si-Slc22a17 and si-negative control using Lipofectamine RNAiMAX Reagent (Invitrogen) and cultured for 24 h. The medium was then refreshed, and recombinant mouse LCN2 was added to the cells at 2 μg/mL 48 h after transfection. After incubation for an additional 24 hours, RNA was isolated and the cell culture supernatant was collected to examine MMP9 protein levels.

### Assessment of cardiomyocyte apoptosis

Death of cardiomyocytes at the border region of the infarcted heart was detected using the DeadEnd^TM^ Fluorometric TUNEL kit (Promega, Madison, WI, USA).

H9C2 cells were treated with conditioned medium from *Card9*-overexpressing and control RAW264.7 cells for 48 h, and then their apoptosis was evaluated using TUNEL staining.

### Enzyme-linked immunosorbent assay (ELISA)

Plasma was separated from ethylenediaminetetraacetic acid (EDTA) anti-coagulated blood samples of mice by centrifugation at 3000 rpm/min for 10 min at 4 °C. Cell culture medium was subjected to a centrifugation at 2000 ×g for 10 min at 4 °C in a refrigerated centrifuge to collect supernatant. LCN2 levels were determined using mouse LCN2 ELISA kits (LCN2 in plasma: R&D Systems, Minneapolis, MN, USA; LCN2 in cell culture supernatant: Abcam, Cambridge, UK). MMP9 levels in the cell culture supernatants were detected using a mouse MMP9 ELISA kit (RayBiotech, Norcross, GA, USA).

### Cardiac cell sorting

Hearts from WT mice 3 days post-MI were perfused with cold PBS, cut into small pieces, and dissociated with collagenase Ia (Sigma-Aldrich) at 37 °C for 30 min to obtain single-cell suspensions, which were then used for fluorescence-activated cell sorting (FACS) and magnetic-activated cell sorting (MACS). For FACS, the cells were incubated with PerCP-Cy5.5-labeled anti-CD45 (550994, BD Biosciences, San Jose, CA, USA) and PE anti-F4/80 (12480182, eBioscience, San Diego, CA, USA) at 4 °C for 30 min in the dark, and the expression of the two molecules was analyzed on FACS Aria III (BD Bioscience). CD45^-^, CD45^+^F4/80^-^, and CD45^+^F4/80^+^ cell populations were collected, and CARD9 protein expression in these populations was assayed with western blotting. For MACS, the cells were first incubated with biotin anti-CD45 (13045182, eBioscience) and sorted using the Release Mouse Biotin Positive Selection Kit (STEMCELL, Seattle, WA, USA) to obtain CD45^-^ and CD45^+^ cells. Subsequently, CD45^+^ cells were incubated with PE anti- F4/80 and sorted using a mouse PE-positive Selection Kit (STEMCELL) to obtain CD45^+^F4/80^-^ and CD45^+^F4/80^+^ cells. *Card9* mRNA level in these cells was evaluated by qPCR.

### Flow cytometric analysis

EDTA anti-coagulated whole blood and cardiac single-cell samples were incubated with PE-CF594 anti-CD45.1 (562452) and V500 anti-CD45.2 (562129), which were purchased from BD Biosciences, for 30 min in the dark. Red blood cells were removed with exposure to the lysis buffer for 1 min. Data were collected with an LSR Fortessa cell analyzer (BD Biosciences).

### Quantitative PCR

Total RNA isolation, complementary DNA synthesis, and qPCR analysis were performed following protocols we previously described.^67^ The expression of genes was normalized to *Gapdh*. The primers are shown in Supplementary Table 2.

### Histopathology

Heart tissues were fixed, embedded and cut into sections as described previously.^33^ Subsequently, the cardiac sections were subjected to various staining procedures: hematoxylin and eosin (H&E) staining for morphological observation, Sirius red and Masson’s trichrome staining for fibrosis and infarct area analysis, and wheat germ agglutinin (Sigma-Aldrich) staining for cellular hypertrophy evaluation. For immunohistochemical analysis, sections were labeled with primary antibodies followed by HRP-linked secondary antibodies. Diaminobenzidine tetrahydrochloride was used for the colorimetric reaction. For immunofluorescence, frozen sections were labeled with primary antibodies followed by Alexa Flour 488- or Alexa Flour 555-conjugated secondary antibodies (Invitrogen). Antibodies against Mac3 (sc-19991), MMP2 (sc-10736), and CARD9 (sc-49408) were purchased from Santa Cruz Biotechnology; anti-CD31 (DIA-310) from Dianova; anti-MMP9 (ab38898 and ab283575), neutrophils (ab2557), F4/80 (ab6640), and SLC22A17 (ab124506) from Abcam; anti-α-actinin (A7811) from Sigma-Aldrich; anti-LCN2 (AF1857) from R&D System. Anti-CD31 was diluted to 1:50, and all other primary antibodies were diluted to 1:100. A Nikon ECLIPSE Ni microscope (Nikon, Japan) or a laser confocal microscope (Leica, Buffalo Grove, IL, USA) were used for image capture. ImagePro Plus 3.0 was used for analysis.

### Western blotting

Protein from tissues and cells were isolated using RIPA buffer. The protein levels of targets were detected using western blotting method as described in our previous study.^33^ Primary antibodies against CARD9 (sc-99054) and MMP2 (sc-10736) were obtained from Santa Cruz Biotechnology; MMP3 (ab52915) and MMP9 (ab38898) from Abcam; cleaved caspase 3 (9661 S), caspase 3 (9662 S), phosphorylated NF-κB p65 (3033 S), and NF-κB p65 (8242 S) from Cell Signaling Technology; GAPDH (TA-08) from ZSGB-Bio. The primary antibodies were all diluted to 1:1000. Infrared fluorescence-labeled secondary antibodies were used with a dilution of 1:10000. The membranes were scanned using an Odyssey Infrared Imaging System (Li-Cor Bioscience, Lincoln, NE, USA).

### Statistics

All data are presented as the mean ± SEM. Two-tailed unpaired *t-*test was performed to compare two groups, and one-way or two-way ANOVA were used for multiple group comparisons; the difference was statistically significant if *p* < 0.05.

### Supplementary information

Supplementary materials

Supplementary materials 2

## Data Availability

The raw data of RNA-Seq have been deposited in NCBI GEO under the accession number GSE235257. The data that support the findings of this study is available from the corresponding author upon reasonable request.
