# Supplementary material for: Macrophage CARD9 mediates cardiac injury following myocardial infarction through regulation of lipocalin 2 expression
Source: Signal Transduct Target Ther. 2023 Oct 13;8:394. doi: 10.1038/s41392-023-01635-w (PMC10570328; doi:10.1038/s41392-023-01635-w)
Supplement: Supplementary file 1 — Supplementary materials [file 41392_2023_1635_MOESM1_ESM.docx]

Supplementary Materials for

Macrophage CARD9 mediates cardiac injury following myocardial infarction via regulation of lipocalin 2 expression

Yan Liu^#^, Yi-hui Shao^#^, Jun-meng Zhang, Ying Wang, Mei Zhou, Hui-qin Li, Cong-cong Zhang, Pei-jie Yu, Shi-juan Gao, Xue-rui Wang, Li-xin Jia, Chun-mei Piao, Jie Du, Yu-lin Li*

Correspondence to: lyllyl_1111@163.com (Yu-lin Li)

# The authors contributed equally to this work.

**This PDF file includes:**

Materials and Methods

Figures. S1 to S12

Tables S1 to S2

**Materials and methods**

**RNA-sequencing and data analysis**

Hearts from WT sham, WT, and *Card9* KO mice 3 days after MI were collected, and total RNA in the cardiac tissues below the ligature was extracted with TRIzol Reagent (Invitrogen). Standard Illumina protocols were used for RNA Library construction. The mRNA was enriched, fragmented, and reverse-transcribed into double-stranded cDNA. End reparation and 3’-end single nucleotide adenine addition were performed. Sequencing adaptors were ligated into fragments, which were then enriched by PCR amplification. RNA libraries were sequenced at 10 pM using the Illumina HiSeq 2000 system at the Beijing Genomics Institute (BGI, Wuhan, China) according to the manufacturer’s instructions. After filtering, the clean reads were mapped to the mm9 mouse whole genome using the BWA/Bowtie2 tool. The fragments per kilobase per million method was used to calculate gene expression. We applied the NOIseq method to screen DEGs between two groups using the following criteria: fold-change ≥ 2.0 and diverge probability ≥ 0.8. GO analysis was performed.

**Analysis of mouse MI scRNA-seq data**

The scRNA seq raw data of mouse cardiac CD45^+^ cells (GSE163465)^1^ was obtained from the GEO database. The CD45^+^ cells isolated from the cardiac ischemic zone of sham and MI mice with a C57BL/6 background^1^. The Seurat R package (version 4.2.0) was used to determine cellular heterogeneity. DEGs were identified using the FindAllMarkers function in Seurat by comparing cells in one cluster with all other cells. A non-parametric Wilcoxon rank-sum test was used in this process.

**Growth factor measurement**

After four hours of exposure to necrotic cardiomyocytes, the culture medium of both WT and Card9 KO BMDM was refreshed andthe cells were then cultured for another 16 hours. After collecting the supernatant, the levels of the various growth factors were checked using a Bio-Plex system (Bio-Rad) according to the manufacturer’s instructions^2^.

**References**

1. Jin, K. *et al.* Single-cell RNA sequencing reveals the temporal diversity and dynamics of cardiac immunity after myocardial infarction. *Small Methods* **6**, e2100752 (2022).
2. Yang, M. *et al.* Tumor cell-activated CARD9 signaling contributes to metastasis-associated macrophage polarization. *Cell. Death. Differ.* **21**, 1290-1302 (2014).

**
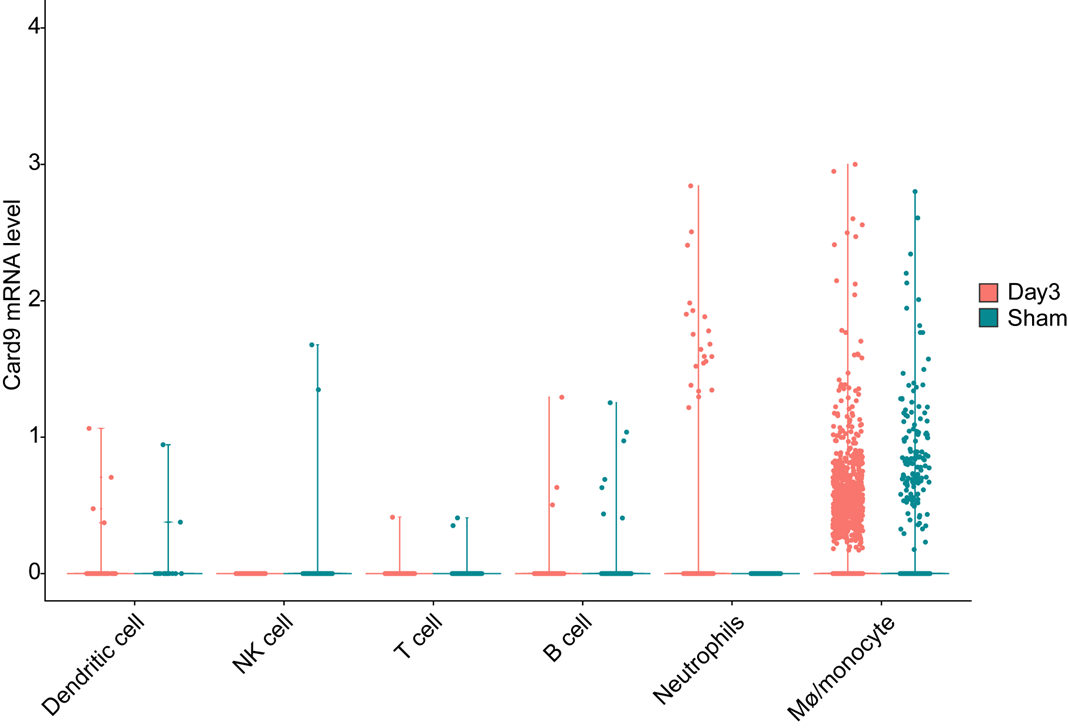
**

**Figure. S1. *Card9* expression in cardiac CD45^+^ cells post-MI by analyzing scRNA-seq dataset GSE163465.** scRNA-seq dataset GSE163465 which was downloaded from GEO database evaluated CD45^+^ cells from the left ventricles of mouse hearts at different time point after MI. Data from sham and 3 days post-MI groups was used to analyze *Card9* expression in each cell population.


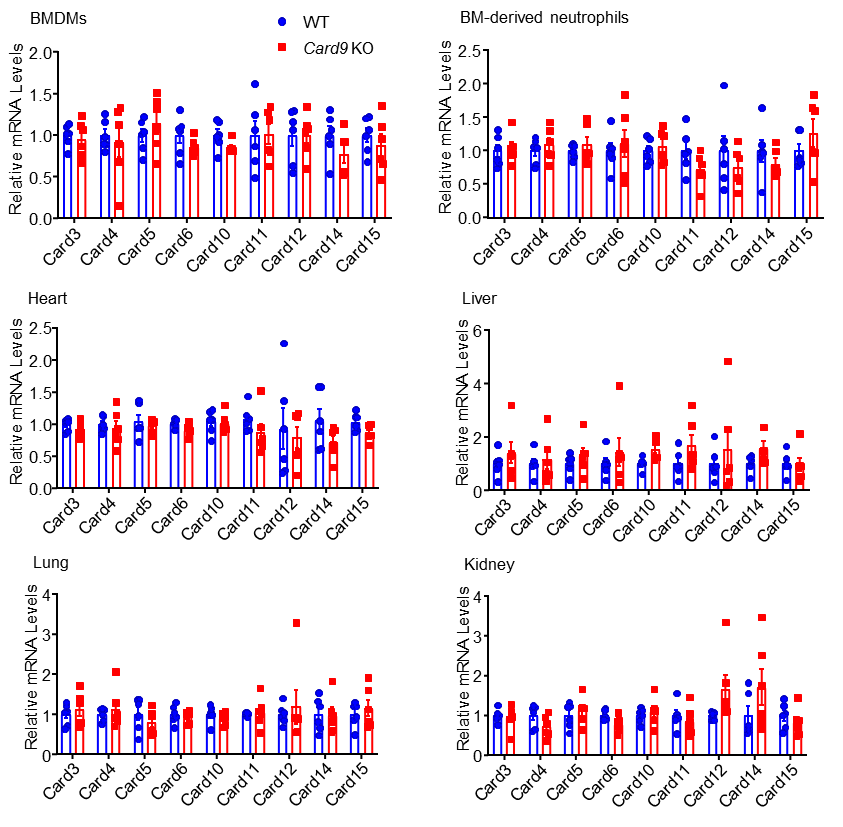


**Figure. S2. *Card9* knockout doesn’t affect other Card family gene expression.** qPCR analysis of the expression of Card family genes in bone marrow-derived macrophages (BMDMs), bone marrow-derived neutrophils, heart, liver, lung, and kidney from WT and *Card9* KO mice (n = 6). Data represent the mean ± SEM of fold induction with respect to WT group after normalization to *Gapdh*.


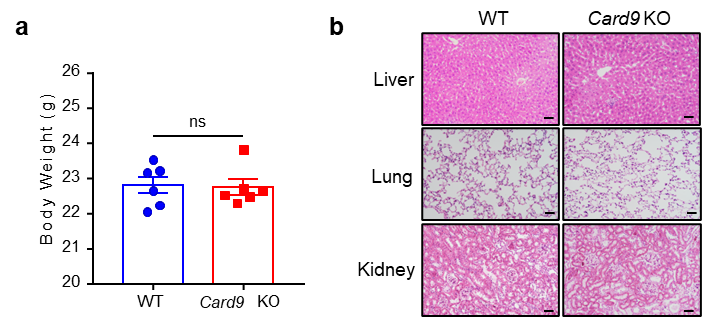


**Figure. S3. *Card9* knockout doesn’t affect body weight and morphology of vital tissues in mice. a** The body weight of 10- to 11-week-old male WT and *Card9* KO mice (n = 6). Data represent the mean±SEM. **b** Representative H&E staining on liver, lung, and kidney tissue sections of 12- to 16-week-old WT and *Card9* KO mice (n = 6). Scale bar: 50μm.

**
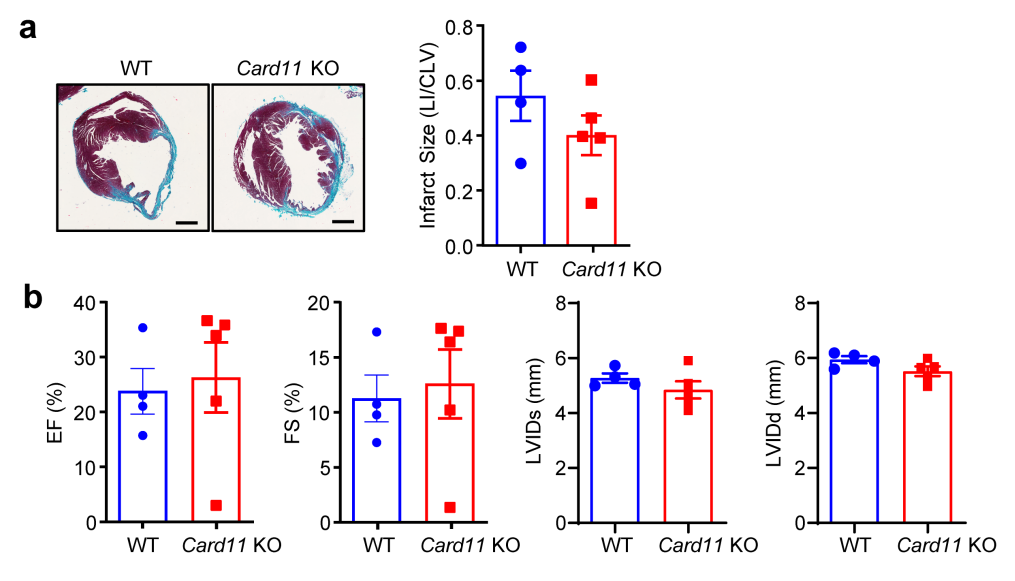
**

**Figure. S4. *Card11* knockout doesn’t affect cardiac function and remodeling post-MI.** Ten- to fourteen-week-old male WT and *Card11* KO mice underwent MI surgery. **a** Representative Masson’s trichrome staining of cardiac sections at 28 days post-MI and measurement of infarct size (n = 4-5). Infarct size is presented as the ratio of infarcted area length to left ventricular circumference. **b** Left ventricular function was assessed by echocardiography. Left ventricular end-systolic internal diameter (LVIDs) and end-diastolic internal diameter (LVIDd) were measured and ejection fraction (EF) and fractional shortening (FS) were calculated. Data represent the mean ± SEM. Scale bar: 1mm.


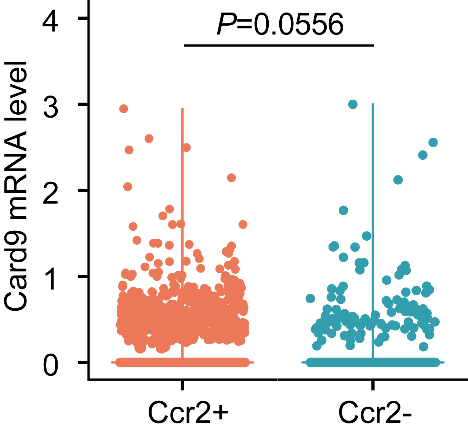


**Figure. S5. *Card9* expression in cardiac CCR2^+^ and CCR2^-^ macrophages post-MI.** Data of 3 days post-MI group from scRNA-seq dataset GSE163465 was used to analyze *Card9* expression in CCR2+ and CCR2- macrophage/monocyte populations.

**
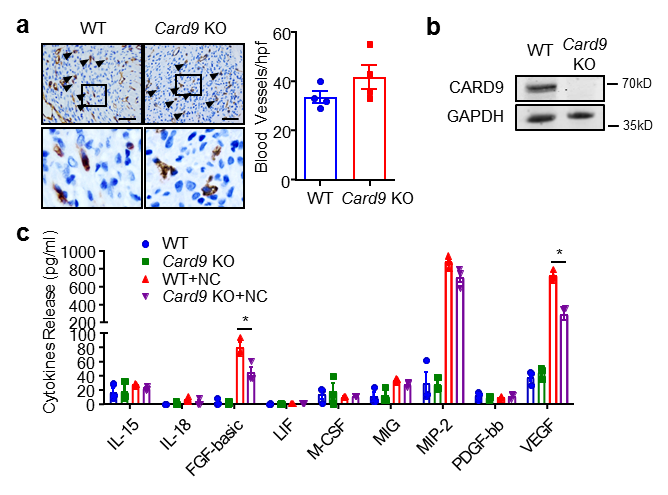
**

**Figure. S6. *Card9* knockout does not affect angiogenesis post-MI. a** Representative Immunohistochemical staining for CD31 in border region of WT and *Card9* KO cardiac sections at 7 days post-MI (n = 4). The capillary density per high power field was qualified. Data represent the mean±SEM. Scale bar: 50μm. **b** Western blot analysis of CARD9 protein in BMDMs isolated from WT and *Card9* KO mice. **c** Growth factors released into culture supernatants from necrotic cell (NC)-treated WT and *Card9* KO BMDMs were determined. Data represent the mean±SEM; three independent experiments were performed. **p*<0.05.


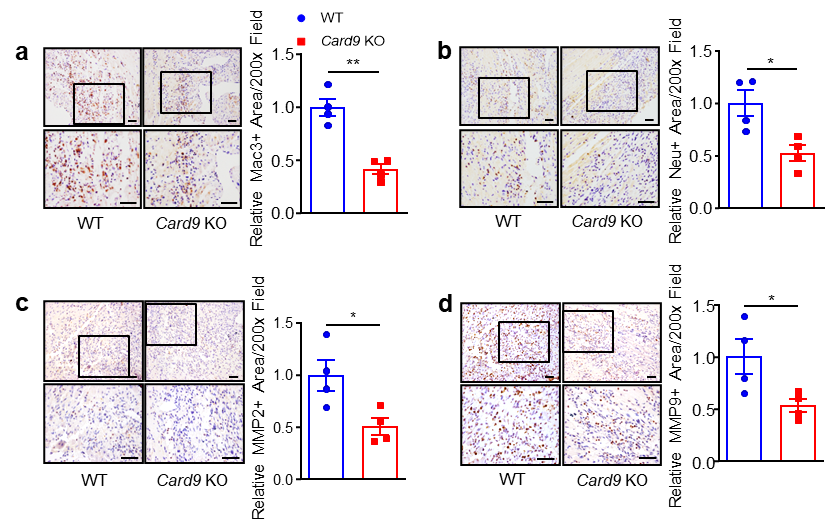


**Figure. S7. *Card9* knockout reduces macrophage and neutrophil infiltration and MMP2 and MMP9 expression post-MI.** Immunohistochemical staining for Mac3 (**a**), Neutrophil (**b**), MMP2 (**c**), and MMP9 (**d**) in border region of WT and *Card9* KO cardiac sections at 3 days post-MI (n = 4). The positive area was qualified and showed as fold induction with respect to WT group. Data represent the mean±SEM. Scale bar: 50μm. **p*<0.05, ***p*<0.01.


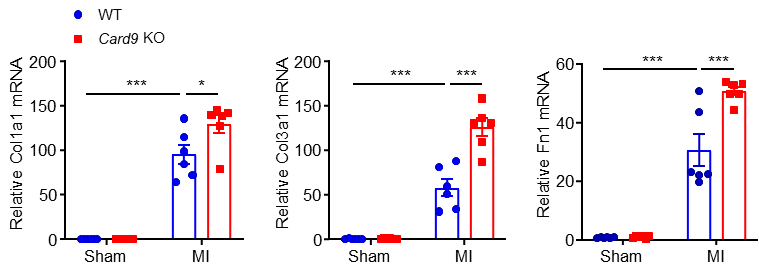


**Figure. S8. *Card9* knockout increases the expression of ECM genes post-MI.** mRNA expression levels of Collagen 1a1 (*Col1a1*), *Col3a1*, and Fibronectin 1 (*Fn1*) were examined by qPCR in the hearts of both WT and *Card9* KO mice at 7 days post-MI (n = 6). Data represent the mean ± SEM of fold induction with respect to WT Sham mice after normalization to *Gapdh*. **p*<0.05, ****p*<0.001.


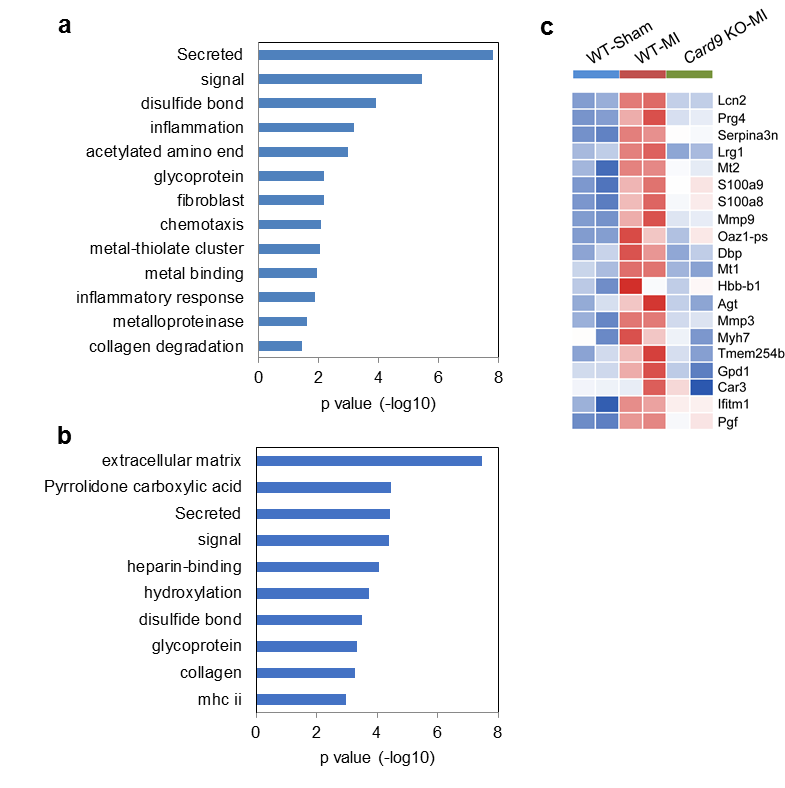


**Figure. S9. GO analysis of differentially expressed genes (DEGs) in the hearts of WT and *Card*9 KO mice post-MI.** RNA-sequencing was performed with mouse cardiac tissue from WT Sham, WT MI and *Card9* KO MI groups (n=2). Down-regulated (**a**) and up-regulated (**b**) genes in *Card9* KO mice compared with WT mice after MI were selected for Gene Ontology (GO) analysis. **c** Hot map of DEGs in the right panel of Fig. 4a ranking from high to low according to the diverge probability.


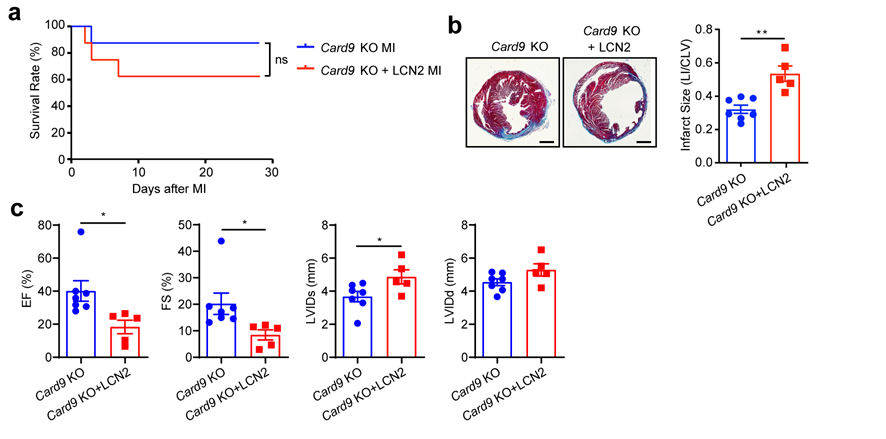


**Figure. S10. LCN2 enhances cardiac injury post-MI in *Card9* KO mice. a** Survival analysis *Card9* KO mice treated with or without recombinant mouse LCN2 post-MI (n = 8)**. b** Representative Masson’s trichrome staining of cardiac sections at 28 days post-MI and measurement of infarct size (n = 5-7). Infarct size is presented as the ratio of infarct area length to left ventricular circumference. **c** LV function was assessed by echocardiography. LVIDs and LVIDd were measured and EF and FS were calculated. Data represent the mean ± SEM. Scale bar: 1mm. ***p* < 0.01.


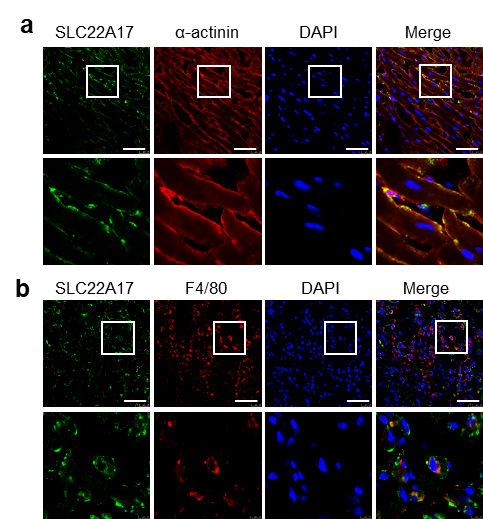


**Figure. S11. LCN2 receptor expresses on cardiomyocytes and macrophages.** Co-staining of LCN2 receptor (SLC22A17) and α-actinin (**a**) or F4/80 (**b**) in the border region of WT hearts at 3 days post-MI. Nuclei were stained with DAPI. Scale bar: 50 μm.


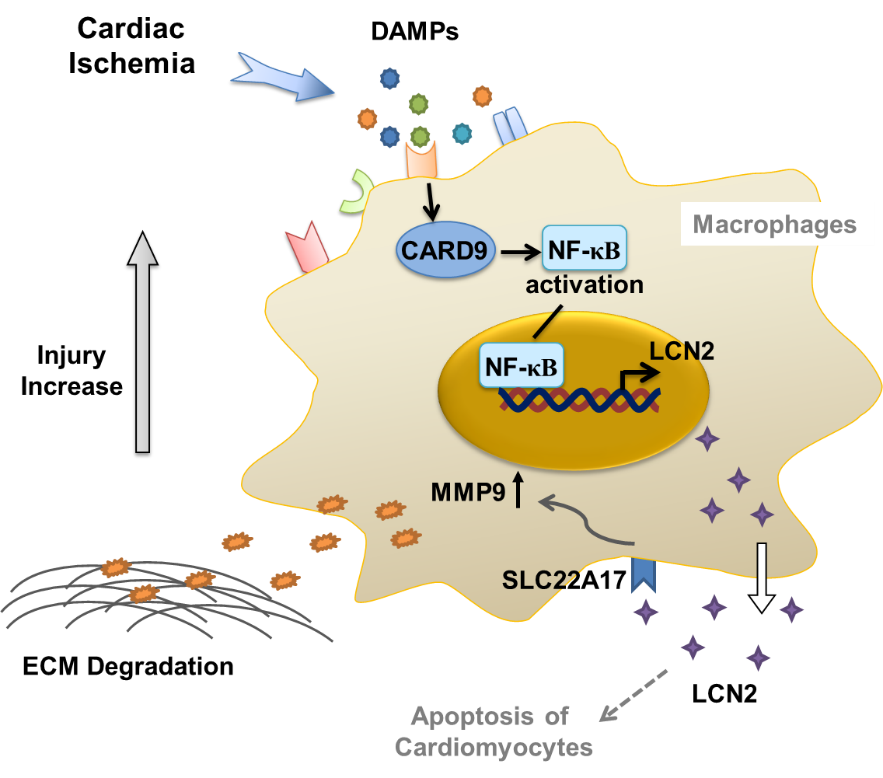


**Figure. S12. Diagram illustrating the mechanism for CARD9 regulating cardiac injury post-MI.** Ischemia induced death of cardiomyocytes and release of DAMPs. Necrotic cells or DAMPs then activated CARD9 signaling in macrophages, which resulted in NF-κB activation, and subsequent upregulated LCN2 expression. LCN2 might enhance further injury post-MI by increasing MMP9 expression and release. DAMPs indicates danger-associated molecular patterns; CARD9, caspase recruitment domain-containing protein 9; NF-κB, nuclear factor kappa-B; LCN2, lipocalin 2; MMP9, metalloproteinase 9; ECM, extracellular matrix. **Table S1. Echocardiographic analysis of WT and *Card9* KO mice at 28 days post-MI.**

|  | **WT Sham (n=7)** | ***Card9* KO Sham (n=6)** | **WT MI (n=6)** | ***Card9* KO MI (n=6)** |
| --- | --- | --- | --- | --- |
| **EF(%)** | 51.88±4.09 | 53.65±5.34 | 23.56±2.81* | 47.11±5.16# |
| **FS(%)** | 26.51±2.56 | 27.33±3.01 | 10.95±1.38* | 23.87±2.91# |
| **LVIDd (mm)** | 4.02±0.24 | 3.98±0.31 | 5.16±0.38* | 4.69±0.35 |
| **LVIDs (mm)** | 2.96±0.23 | 2.94±0.29 | 4.62±0.35** | 3.61±0.25# |
| **LVAWd (mm)** | 1.37±0.1323 | 1.33±1.12 | 0.98±0.10* | 1.12±0.08 |
| **LVAWs (mm)** | 1.69±0.14 | 1.86±1.11 | 1.27±0.16 | 1.68±0.05# |
| **LVPWd (mm)** | 0.83±0.07 | 1.10±0.15 | 0.80±0.09 | 0.85±0.18 |
| **LVPWs (mm)** | 1.18±0.09 | 1.31±0.17 | 0.90±0.09 | 0.97±0.21 |

**P* < 0.05, ***P* < 0.01 (versus WT Sham); #*P* < 0.05 (versus WT MI)

EF indicates ejection fraction; FS, fractional shortening; LVIDd, left ventricular end-diastolic internal diameter; LVIDs, left ventricular end-systolic internal diameter; LVAWd, left ventricular end-diastolic anterior wall thickness; LVAWs, left ventricular end-systolic anterior wall thickness; LVPWd, left ventricular end-diastolic posterior wall thickness; LVPWs, left ventricular end-systolic posterior wall thickness.

**Table S2. Primers used in this study.**

| **Gene** | **Forward** | **Reverse** |
| --- | --- | --- |
| ***Card3*** | aaatcatcccccacaggag | ggtccaggagaaccagtgtt |
| ***Card4*** | tttaagggtgaagccaaagg | ggcagacaaatcaggattcag |
| ***Card5*** | gagcagctgcaaacgactaa | gtccacaaagtgtcctgttctg |
| ***Card6*** | attttgtggccctccaaga | ttgaaagtgggcaacatgg |
| ***Card9*** | ctctgtgcaggagggtaagc | tccgtagggagaagatggtg |
| ***Card10*** | tgcagggcgagctacagt | gcagatcctccatctcttgc |
| ***Card11*** | tctccagagcgagtttcttctt | tgttttctgaccggctgac |
| ***Card12*** | tgatctccaagagatgaagttgg | gatcaaattgtgaagattctgtgc |
| ***Card14*** | gagaaactccgctccatgac | cctcatccagactctgttcca |
| ***Card15*** | tgtggagtcaccgcaaaac | tcctctgtgcctggaactct |
| ***Mmp2*** | cgatgtcgcccctaaaacag | gcatggtctcgatggtgttc |
| ***Mmp3*** | gtgtgtggttgtgtgctcat | ttccctgtcatctccaaccc |
| ***Mmp9*** | tgggcgttagggacagaaat | gaaccataacgcacagaccc |
| ***Lcn2*** | ggccagttcactctgggaaa | ccacactcaccacccattca |
| ***Slc22a17*** | ctgtttctggagtctgcacg | gcaaaggaaaaggtggacgt |
| ***Col1a1*** | gagcggagagtactggatcg | gttcgggctgatgtaccagt |
| ***Col3a1*** | cccctggttcttctggacat | tgggcctttgatacctggag |
| ***Fn1*** | tcccgggcagaaagtacatt | ttcagggaggttgagctctg |
| ***Gapdh*** | aatgcatcctgcaccacc | atgccagtgagcttcccg |
