# Supplementary material for: Macrophage CARD9 mediates cardiac injury following myocardial infarction through regulation of lipocalin 2 expression
Source: Signal Transduct Target Ther. 2023 Oct 13;8:394. doi: 10.1038/s41392-023-01635-w (PMC10570328; doi:10.1038/s41392-023-01635-w)

Uncropped blots

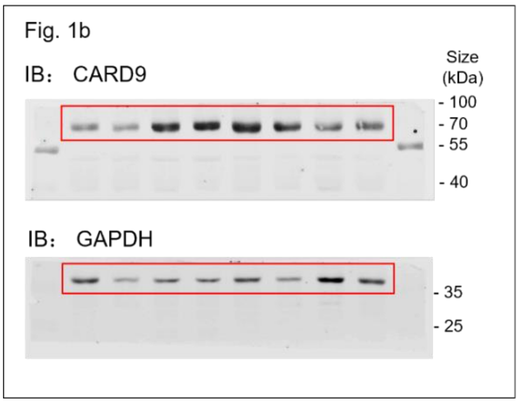

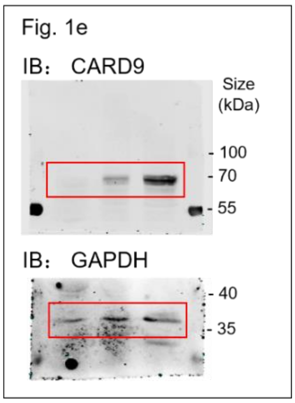

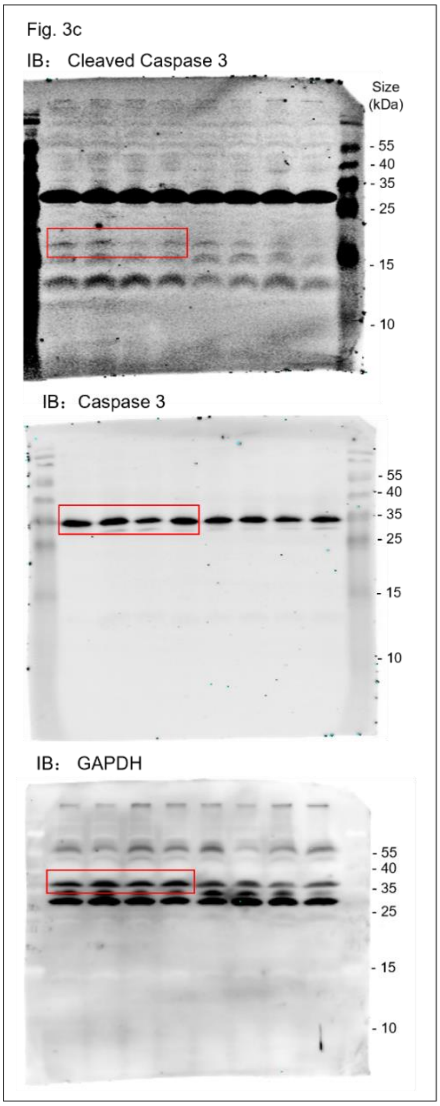

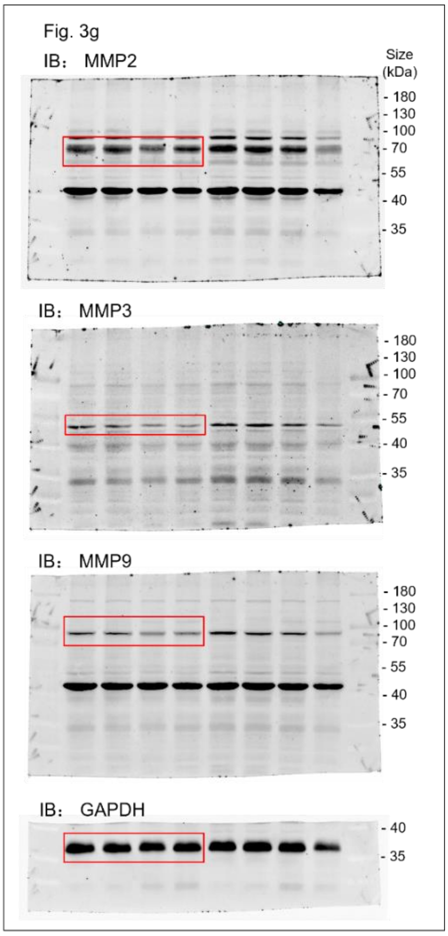

Fig. 5a

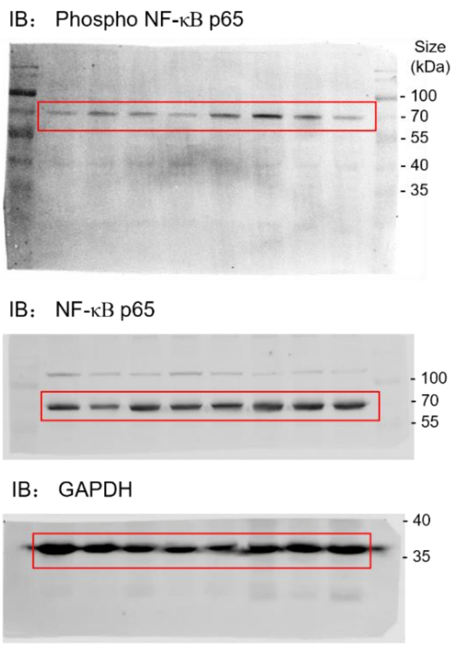

Fig. 5b

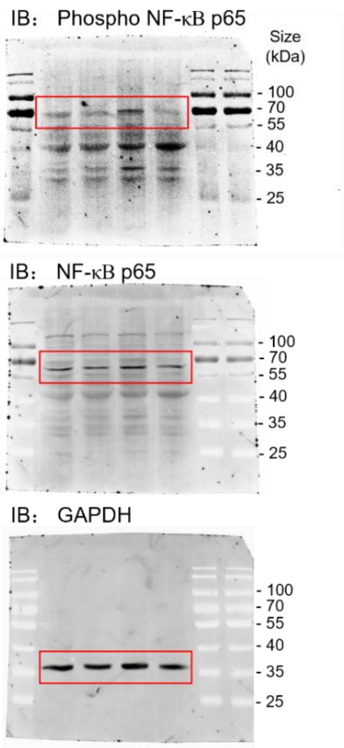

Supplementary Fig. 5b

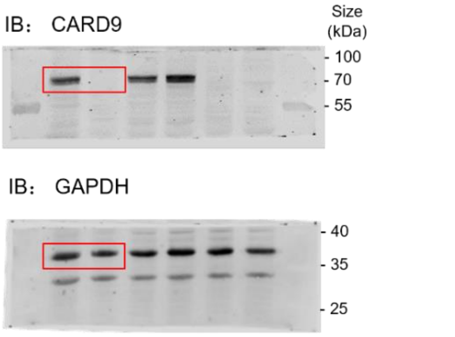

Supplement: Supplementary file 2 — Supplementary materials 2 [file 41392_2023_1635_MOESM2_ESM.pdf]
